# Lactylation: a novel driver of drug resistance in the tumor microenvironment

**DOI:** 10.20517/cdr.2025.90

**Published:** 2025-08-04

**Authors:** Chunwei Li, Ziqiang Liu, Dezheng Kong, Zhengze Li, Lifeng Li

**Affiliations:** ^1^National Engineering Laboratory for Internet Medical Systems and Applications, The First Affiliated Hospital of Zhengzhou University, Zhengzhou 450052, Henan, China.; ^2^The First Affiliated Hospital of Zhengzhou University, Zhengzhou 450001, Henan, China.; ^3^Cancer Center, The First Affiliated Hospital of Zhengzhou University, Zhengzhou 450052, Henan, China.; ^4^Fuwai Central China Cardiovascular Hospital, Zhengzhou 451460, Henan, China.; ^#^These authors contributed equally to this work and share first authorship.

**Keywords:** Lactylation, epigenetic, drug resistance, tumor microenvironment

## Abstract

Lactylation, a novel lactate-derived lysine post-translational modification (PTM), has emerged as a critical epigenetic regulator driving drug resistance within the tumor microenvironment (TME). This review systematically delineates the enzymatic underpinnings of lactylation, its induction via the glycolysis-lactate axis influenced by key TME features (hypoxia, inflammation), and its multifaceted roles in promoting resistance. Specifically, lactylation orchestrates transcriptional reprogramming of resistance-associated genes (e.g., oncogenes, immune checkpoints, epithelial–mesenchymal transition factors), enhances DNA damage repair capacity (e.g., via NBS1/MRE11 lactylation), activates pro-survival autophagy, and modulates immunosuppressive signaling pathways (e.g., PI3K/AKT, NF-κB, JAK/STAT). Furthermore, it facilitates critical resistance phenotypes including immune evasion, metastasis, and angiogenesis. The review summarizes emerging therapeutic strategies targeting lactylation, such as inhibition of lactate production (LDHA/LDHB), lactate transport (MCT1/4), lactyltransferases (e.g., p300), or downstream effectors, highlighting their potential to overcome multifactorial resistance. However, elucidating the context-dependent roles, crosstalk with other PTMs, and developing specific inhibitors remain crucial for translating these insights into effective clinical interventions against resistant tumors.

## INTRODUCTION

Cancer progression represents an evolutionary adaptation process where tumor cells dynamically reprogram their epigenetic landscape to overcome microenvironmental stressors. The Warburg effect, a hallmark metabolic shift favoring aerobic glycolysis, generates substantial lactate accumulation that transcends its traditional role as a metabolic byproduct. Emerging insights reveal lactate functions as an epigenetic modulator through lactylation - a novel post-translational modification (PTM) where lactate-derived acyl groups covalently attach to lysine residues on histones and regulatory proteins. This lactylation-driven reprogramming establishes self-reinforcing circuits across three oncogenic axes: metabolic plasticity, metastatic competence, and immune evasion^[1-3]^.

Histone H3K9la/H3K18la modifications activate oncogenic transcriptional programs (VCAM1, LAMCS2, ESM1). These modifications override growth restriction checkpoints and coordinate with PI3K/AKT-mTOR and Hippo pathways to sustain proliferative signaling. Hypoxia-lactylation crosstalk further induces epithelial–mesenchymal transition (EMT) through Sox9/CENPA overexpression and extracellular matrix remodeling, creating pre-metastatic niches via lactylated MOESIN-mediated cytoskeletal reorganization^[4-6]^. Concurrently, lactate reshapes the tumor immune landscape through dual-phase macrophage polarization - suppressing M1 phagocytic activity via IL-6/ARG1/CCL5 downregulation while enhancing M2 pro-tumorigenic functions through CCL18 induction. This immunosuppressive microenvironment is further consolidated through TGF-β/IL-10-mediated Treg expansion and PD-1/CTLA-4 checkpoint activation^[7-9]^.

Critically, these interconnected pathways converge on STAT3 survival signaling. This convergence explains the frequent failure of conventional therapies targeting isolated pathways. This functional convergence provides a compelling explanation for the frequent failure of conventional therapies targeting isolated pathways, as lactylation enables tumors to bypass such targeted assaults through compensatory mechanisms. Therefore, the discovery of lactylation as a central epigenetic integrator within the tumor microenvironment (TME) offers a transformative mechanistic framework not only for deconvoluting the multifactorial nature of treatment resistance but also for identifying novel therapeutic vulnerabilities. Pharmacological inhibition of lactate transporters (MCT1/4) combined with epigenetic modifiers targeting lactylation enzymes may disrupt these adaptive circuits, potentially restoring chemosensitivity and immune surveillance. Ongoing clinical trials exploring lactylation-directed therapies (NCT05163505, NCT04889716) underscore the translational potential of this paradigm-shifting mechanism^[1,4,7]^.

## MECHANISMS OF LACTYLATION-MEDIATED THERAPEUTIC RESISTANCE IN SOLID TUMORS

### Enzymatic regulators of lactylation in tumor drug resistance

Lactylation, a recently identified PTM, has emerged as a critical mediator of tumor drug resistance. Its establishment and regulation within the TME are fundamentally governed by a network of metabolic and epigenetic enzymes. These enzymes act as critical initiators and amplifiers of the lactylation cascade, either by directly catalyzing the lactylation reaction or by influencing lactate availability and histone accessibility. This enzymatic network thereby directly links key microenvironmental cues to the development of drug resistance phenotypes through lactylation. These enzymes regulate both lactylation levels and key drug resistance pathways. These pathways include DNA repair, EMT, immune escape, and metabolic adaptation^[10]^. While these enzymatic pathways illustrate the metabolic-epigenetic link to resistance, it is imperative to dissect the dynamic regulation of lactylation. The balance between lactyltransferases (e.g., p300, AARS1) and putative delactylases, as well as lactate flux dictated by microenvironmental cues (hypoxia, inflammation), critically determines functional outcomes. Overlooking this dynamism risks oversimplifying lactylation’s contribution to resistance phenotypes.

### LDHA: a central driver of lactate supply and resistance phenotypes

Lactate dehydrogenase A (LDHA) catalyzes the conversion of pyruvate to lactate, thus supplying the metabolic substrate for histone lactylation. Elevated LDHA expression has been consistently observed in breast, lung, and ovarian cancers and is strongly associated with resistance to platinum-based chemotherapies and radiotherapy^[11-13]^. A pivotal study demonstrated that LDHA-mediated lactylation of NBS1 at K388 enhances homologous recombination (HR) repair capacity. This modification reduces cisplatin sensitivity in gastric cancer cells^[14]^. These findings suggest LDHA not only fuels lactylation but also directly modulates drug resistance mechanisms, making it a dual-function therapeutic target and predictive biomarker^[15]^.

### ALDOB–PDK1–LDHB–CEACAM6 axis in colorectal cancer

A study from Taiwan revealed that elevated expression of aldolase B (ALDOB) in CRC activates pyruvate dehydrogenase kinase 1 (PDK1), thereby increasing lactate production. The elevated lactate stimulates LDHB expression in neighboring cells, which in turn enhances CEACAM6 activity - a key driver of cell adhesion and chemoresistance. This metabolic axis promotes resistance to conventional chemotherapies such as 5-fluorouracil (5-FU) and oxaliplatin^[16]^.

### AKR1B10–LDHA–H4K12la axis in lung adenocarcinoma

As an aldo-keto reductase family member, AKR1B10 upregulates LDHA expression. Consequently, it significantly increases histone H4K12 lactylation (H4K12la). Elevated H4K12la levels correlate with resistance to pemetrexed (PEM) and cisplatin and are associated with aggressive tumor progression. Clinical monitoring of AKR1B10 and H4K12la may enable personalized resistance risk stratification^[17]^.

### PFK-1–ZEB1 axis in bladder cancer

Phosphofructokinase-1 (PFK-1), a rate-limiting enzyme in glycolysis, is critical for maintaining lactate-driven histone lactylation. Impaired PFK-1 activity reduces glycolytic flux and histone lactylation, leading to downregulation of the EMT-associated transcription factor ZEB1. This, in turn, suppresses invasion and chemoresistance to gemcitabine in bladder cancer cells^[18]^. Thus, PFK-1 and lactylation status may serve as predictive biomarkers of therapeutic response.

### ALDH1A3–PKM2–lactate axis in glioblastoma

Moreover, studies in glioblastoma (GBM) revealed that aldehyde dehydrogenase 1A3 (ALDH1A3) interacts with pyruvate kinase M2 (PKM2), enhancing its tetramerization and thus promoting lactate production. This process increases lactylation modification levels, thereby enhancing the resistance of GBM stem cells to temozolomide (TMZ) chemotherapy^[19]^. Clinically, the activities of ALDH1A3 and PKM2 may serve as important biomarkers for predicting therapeutic responses and assessing drug resistance risk in GBM patients.

### AARS1: a lactylation-driven therapeutic target in cancer resistance

Alanyl-tRNA synthetase 1 (AARS1) has recently been identified as a noncanonical lactyltransferase that directly catalyzes the lactylation of key resistance-related proteins. AARS1-mediated lactylation targets include: p53, reducing pro-apoptotic signaling^[20]^, YAP–TEAD transcription complex and cGAS, disrupting antitumor innate immunity^[21]^. This enzyme serves as a dual lactate sensor and effector, mediating metabolic reprogramming and therapy resistance. AARS1 is overexpressed is gastric cancer and hepatocelluar carcinoma (HCC), and associates with resistance to immune checkpoint inhibitors and targeted therapies. Consequently, AARS1 represents a promising therapeutic target and prognostic biomaker in gastrointestinal cancers. These findings highlight the critical role of enzymatic regulators in lacylation-mediated resistance mechanisms, which are systematically categorized in [Table 1].

**Table 1:** Enzymes/factors mediating lactylation-induced drug resistance in tumors

**Enzyme/factor**	**Downstream target**	**Associated tumor(s)**	**Concrete mechanism**
LDHA	NBS1	Breast, ovarian cancer	Inhibition of LDHA reduces lactate production, thereby inhibiting NBS1 lactylation, impairing DNA repair, and overcoming cancer cell resistance to chemotherapy^[14]^
AKR1B10	H4K12la (histone)	Lung adenocarcinoma	AKR1B10 enhances glycolysis by upregulating LDHA expression. The resulting increase in lactate promotes H4K12la, which activates transcription of the cell cycle-related gene *CCNB1*, contributing to acquired resistance to PEM in brain metastases of lung cancer^[17]^
ALDOB–PDK1–LDHB axis	CEACAM6	Colorectal cancer	ALDOB activates PDK1, which promotes lactate secretion. Lactate-induced CEACAM6 expression enhances resistance to 5-FU in colorectal cancer cells^[16]^
AARS1	p53/YAP/cGAS	Liver, gastric cancer	AARS1 and AARS2 act as intracellular lactate receptors and lactyltransferases, mediating the lactylation of p53, YAP-TEAD, and cGAS. These modifications promote tumorigenesis and development^[20-22]^, although their specific roles in drug resistance require further investigation
KCNK1–LDHA	H3K18la → LDHA self-expression	Breast cancer	KCNK1 binds to and activates LDHA, enhancing glycolysis and lactate production. This promotes H3K18la, which upregulates LDHA expression itself, forming a malignant positive feedback loop^[23,24]^
GLUT3–LDHA	Histone H3 lactylation	Gastric cancer	GLUT3 promotes metastasis and invasion by regulating histone H3 lactylation in gastric cancer cells^[25]^, although its role in drug resistance remains to be clarified
NUSAP1	LDHA expression	Pancreatic cancer	NUSAP1 interacts with c-Myc and HIF-1α to form a transcriptional complex localized at the LDHA promoter, enhancing its expression and increasing glycolysis and lactate production. In turn, lactate stabilizes NUSAP1 protein by preventing its degradation through lysine lactylation (Kla)^[26]^, establishing a positive feedback loop
NSUN2/YBX1	ENO1 → H3K18la	Colorectal cancer	NSUN2 and YBX1 increase lactate production by modifying ENO1 via m5C methylation. Lactate-dependent H3K18la then activates NSUN2 transcription^[27]^, reinforcing a positive feedback loop that drives colorectal cancer progression

Table 1 consolidates key enzymatic regulators and molecular axes identified as drivers of lactylation-mediated drug resistance across diverse tumor contexts. This compilation underscores the breadth of mechanisms by which lactylation is induced (e.g., via glycolytic enzymes like LDHA, PFK-1) or directly catalyzed (e.g., by lactyltransferases like p300, AARS1), and the specific downstream targets (e.g., DNA repair proteins like NBS1, transcription factors, histones) whose functional alteration (e.g., enhanced activity, stability, transcriptional activation) ultimately confers resistance to various therapeutic modalities. The referenced studies provide mechanistic evidence supporting the causal role of these lactylation events in the observed resistance phenotypes. LDHA: Lactate dehydrogenase A; PEM: pemetrexed; ALDOB: aldolase B; PDK1: pyruvate dehydrogenase kinase 1; LDHB: Lactate dehydrogenase B; 5-FU: 5-fluorouracil.

### Glycolysis–lactate–lactylation axis in tumor drug resistance

Building upon the enzymatic foundation, a central driver of lactylation-mediated resistance is the self-reinforcing loop fueled by the Warburg effect. Elevated glycolytic flux generates lactate, which serves as both the substrate for lactylation and a signaling molecule that further enhances glycolysis and lactylation through epigenetic reprogramming. Glycolysis plays a pivotal role in sustaining tumor growth and drug resistance by producing lactate, which serves as a substrate for histone lactylation. This lactylation promotes transcription of oncogenic and resistance-related genes. It thereby forms a self-reinforcing feedback loop that supports metabolic rewiring, invasiveness, and therapy evasion^[2]^.

### Lactate-induced lactylation promotes oncogene expression and feedback activation

Increased glycolytic flux elevates intracellular lactate levels, directly enhancing histone lactylation at specific residues such as H3K18la. This modification promotes the transcription of glycolytic enzymes, thereby reinforcing lactate production.

In breast cancer, KCNK1 upregulates LDHA activity and promotes H3K18la enrichment, which in turn increases *LDHA* gene expression and downstream oncogenes, forming a KCNK1–LDHA–H3K18la–LDHA positive feedback loop that drives metastasis and chemoresistance^[23,24]^. Similarly, in gastric cancer, overexpression of GLUT3 enhances LDHA-mediated lactate production and H3 lactylation, contributing to reduced chemotherapy sensitivity^[25]^.

### Stabilization of glycolysis-enhancing proteins via lactylation

Lactylation also stabilizes key glycolysis-promoting proteins, amplifying metabolic reprogramming. In pancreatic ductal adenocarcinoma (PDAC), lactylation inhibits the degradation of NUSAP1, which enhances glycolytic activity and further stimulates LDHA expression. This NUSAP1–LDHA–lactate axis creates a feed-forward loop that promotes tumor progression and resistance to gemcitabine^[26]^. In lung cancer, lactylation of the insulin-like growth factor 1 receptor (IGF1R) increases its stability and downstream signaling, thereby enhancing lactate generation and sustaining oncogenic metabolism^[28]^.

### Epitranscriptomic modulation of glycolysis–lactylation loops

RNA-modifying enzymes also contribute to lactylation-driven resistance through transcriptional control of glycolysis. In colorectal cancer, NSUN2 and YBX1 enhance ENO1 transcription via m5C RNA modification. The resulting increase in lactate promotes H3K18la enrichment, which in turn upregulates NSUN2, forming a NSUN2–ENO1–lactate–H3K18la feedback loop that supports proliferation and chemoresistance^[27]^.

### Therapeutic implications

The interplay between glycolysis and lactylation establishes a robust feed-forward metabolic-epigenetic loop that is conserved across multiple tumor types. Clinically, elevated levels of LDHA, GLUT3, KCNK1, H3K18la, and lactate may serve as composite biomarkers for drug resistance risk. Therapeutically, targeting components of the glycolysis–lactate–lactylation axis - such as LDHA inhibitors, lactate transport blockers, or lactylation-modifying agents - may help dismantle these self-reinforcing loops and re-sensitize tumors to conventional treatments.

### Inflammatory cytokine-mediated lactylation drives tumor drug resistance

Beyond intrinsic metabolic reprogramming, the inflammatory milieu characteristic of many tumors constitutes a potent extrinsic inducer of lactylation. Inflammatory cytokines play a pivotal role in the development of tumor cell drug resistance by regulating lactylation levels within the TME^[29,30]^. Numerous studies have shown that inflammatory cytokines, lactate metabolism, and lactylation form a close regulatory network in inflammatory diseases and cancer^[31-34]^. In patients with rheumatoid arthritis (RA), the inflammatory microenvironment promotes aerobic glycolysis, resulting in an increase in peripheral blood lactate levels, which in turn drives histone and non-histone lactation modifications, exacerbating the inflammatory response^[35]^. A similar mechanism is more pronounced in the TME: tumor-associated macrophages (TAMs) are stimulated by proinflammatory factors such as IL-6 and TNF-α to upregulate LDHA expression through HIF-1α, resulting in lactate accumulation^[36]^. These lactates activate the epigenetic inheritance of EGFR by mediating lactation of the H3K18 promoter and promote acquired resistance to EGFR-TKI in NSCLC^[37]^.

### Cytokine-induced glycolysis enhances lactate-driven lactylation

Proinflammatory cytokines stimulate glycolytic metabolism in tumor and immune cells, increasing intracellular lactate accumulation and enhancing substrate availability for lactylation.

For example, IL-6 activates the JAK/STAT3 signaling pathway and upregulates histone acetyltransferases such as P300, thereby enhancing histone H3K18 lactylation and resistance-associated gene expression. For instance, histone H3K18 lactation levels in peripheral blood mononuclear cells of patients with sepsis were significantly positively correlated with serum IL-6 and IL-10 concentrations, while enhanced H3K18la modification promoted the expression of genes such as *Arg1* in the late stages of M1 macrophage polarization^[38]^. This process is closely related to cancer progression, but the relationship with tumor drug resistance still needs to be further demonstrated. In colorectal tumors, IL-6 signaling promotes lactylation-mediated repression of retinoic acid receptor gamma (RARγ) in macrophages. This repression triggers the TRAF6–NF-κB axis, further amplifying IL-6 secretion. Such positive feedback reinforces both lactylation and immune-driven resistance^[4]^.

### Direct induction of lactylation by inflammatory signaling

Beyond metabolic reprogramming, certain cytokines directly promote lactylation of resistance-associated proteins. TNF-α induces lactylation of the transcription factor Sox10, enhancing its transcriptional activity and driving resistance and invasiveness in breast cancer cells^[39]^. These examples suggest that inflammatory signals not only create a lactate-rich environment but also actively modulate chromatin states via protein lactylation. Researchers from Guangzhou have demonstrated that activation of PDGF receptor β signaling promotes histone lactylation in clear cell renal cell carcinoma (ccRCC), correlating with treatment resistance and poor patient prognosis^[40]^.

### Clinical implications and therapeutic outlook

The inflammation–lactylation axis constitutes a critical link between immune dysregulation and therapy resistance. Biomarkers such as serum IL-6, H3K18la, and lactylated transcription factors may help stratify patients at high risk for drug resistance. Targeted inhibition of cytokine signaling pathways (e.g., JAK/STAT3 or NF-κB), in combination with lactylation-blocking agents (e.g., P300 inhibitors or LDHA inhibitors), represents a promising therapeutic avenue to interrupt this resistance circuit.

### Hypoxia-induced lactylation and drug resistance in the TME

Hypoxia, a pervasive and hallmark feature of the TME, profoundly reprograms tumor metabolism and epigenetics. Critically, accumulating evidence demonstrates that hypoxia acts as a potent inducer of lactylation modifications, primarily through activation of the HIF-1α signaling axis. This hypoxia-driven lactylation, in turn, promotes drug resistance via multiple downstream cellular pathways^[41-45]^.

Under hypoxic conditions, sustained activation of HIF-1α and c-Myc upregulates LDHA, boosting intracellular lactate production. This elevation in lactate levels provides abundant substrate for histone and non-histone lactylation. Concurrently, HIF signaling upregulates lactylation-related enzymes including P300/CBP and HDACs. This upregulation amplifies lactylation dynamics^[46-49]^.

In breast cancer, HIF-1α induces NBS1 lactylation at lysine 388, enhancing HR-mediated DNA repair and conferring resistance to platinum-based chemotherapy and radiotherapy^[14]^.

### Hypoxia-driven lactylation activates pro-resistance signaling networks

Hypoxia promotes lactylation of several oncogenic effectors, driving signaling cascades that reinforce stemness, invasiveness, and resistance:

In colorectal cancer, lactylation of β-catenin activates the Wnt pathway, enhancing stem-like traits and reducing oxaliplatin sensitivity^[49]^. In non-small cell lung cancer (NSCLC), SOX9 lactylation increases glycolytic flux and promotes an invasive, drug-resistant phenotype^[50]^. In esophageal squamous cell carcinoma (ESCC), hypoxia-induced lactylation of H3K9la and Axin1 contributes to tumor progression and resistance via chromatin remodeling and Wnt pathway modulation^[51-53]^.

These findings support a unifying model in which hypoxia-induced lactylation acts as an epigenetic integrator of metabolic and signaling adaptations that drive therapeutic resistance.

Mitochondria, essential organelles responsible for cellular energy production, are also closely associated with lactylation under hypoxic conditions. Studies have demonstrated that hypoxia induces lactylation of mitochondrial proteins, thereby suppressing oxidative phosphorylation and reducing cellular metabolic activity. While this metabolic reprogramming may temporarily inhibit tumor growth and dissemination^[54]^, in the long term, lactation-mediated inhibition of mitochondrial function promotes tumor cells to adapt to microenvironmental stresses, leading to resistance to chemotherapy, targeted therapy, and even immunotherapy^[55,56]^.

### Therapeutic implications and biomarker perspectives

**Figure 1:**
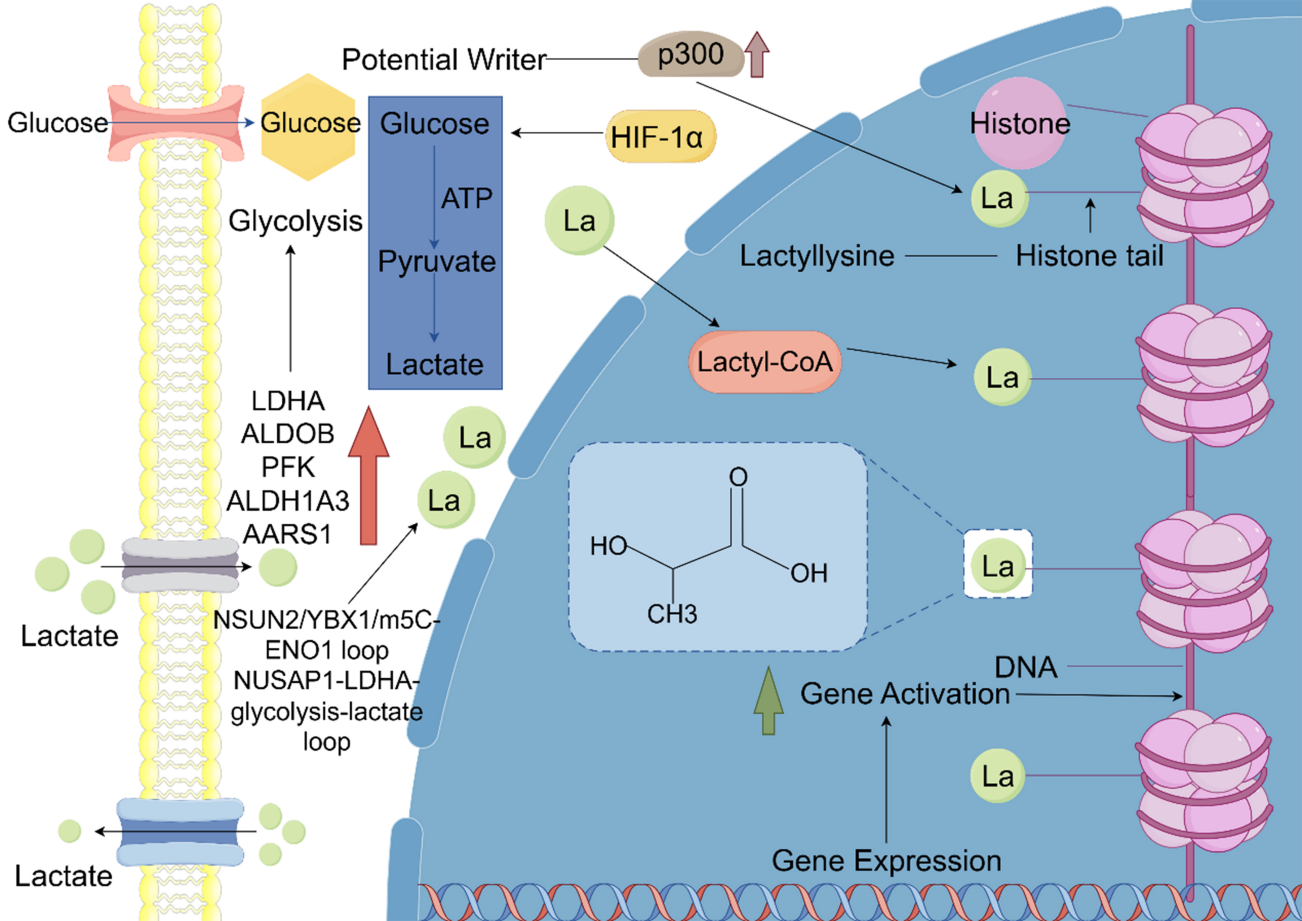
Glucose is metabolized through glycolysis to produce lactate, which can subsequently form Lactyl-CoA. Lactyl-CoA facilitates the conjugation of La to lysine residues on histones, resulting in histone lactylation. This process is catalyzed by p300, a histone acetyltransferase that promotes the addition of lactyl groups to lysine residues on histones. The lactylation of histones alters chromatin structure, enabling transcription factors to more efficiently access DNA, thereby enhancing transcription and gene expression. Histone lactylation has been implicated in gene activation by modifying chromatin architecture to promote transcription. This mechanism likely underpins the role of lactylation in adaptive cellular responses to metabolic stress.

The hypoxia–lactate–lactylation axis represents a critical targetable vulnerability in resistant tumors. Clinical assessment of lactylation markers such as H3K9la, H3K18la, and lactylated β-catenin or NBS1 may guide patient stratification. Therapeutically, interventions that alleviate hypoxia (e.g., anti-angiogenic therapy), inhibit LDHA activity, or target lactylation-modifying enzymes (e.g., P300 inhibitors) could disrupt this resistance mechanism and enhance treatment efficacy. Cellular lactylation modification is shown in Figure 1.

## IMPAIRED DRUG UPTAKE, ENHANCED EFFLUX, AND TARGET ALTERATION MEDIATED BY LACTYLATION

Tumor resistance to chemotherapy is often initiated through fundamental pharmacokinetic alterations, including reduced drug uptake, increased efflux via membrane transporters, impaired prodrug activation, detoxification enhancement, and structural changes in drug targets. Emerging evidence suggests that lactylation participates in modulating these processes, thereby contributing to the early stages of chemoresistance development.

### Reduced drug uptake and increased efflux

Multiple ATP-binding cassette (ABC) transporters - such as P-glycoprotein (ABCB1), MRP1 (ABCC1), and BCRP (ABCG2) - play central roles in mediating drug efflux. Recent proteomic studies revealed that histone H3K18 lactylation enhances the transcription of ABCB1 and ABCC1 in colorectal and HCCs, leading to increased efflux of doxorubicin and paclitaxel. In addition, in NSCLC, lactylation has been associated with elevated expression of solute carrier transporters (e.g., SLC22A1), thereby modulating drug uptake profiles and limiting intracellular drug accumulation^[50,57,58]^.

### Prodrug activation and detoxification

Lactylation also affects enzymes responsible for prodrug activation and drug detoxification. In HCC, histone lactylation promotes upregulation of carboxylesterase 1 (CES1) and aldehyde dehydrogenases (ALDHs), accelerating the hydrolysis of irinotecan and the detoxification of cyclophosphamide metabolites, respectively. Such effects reduce the cytotoxic efficiency of these agents and facilitate resistance. Similarly, lactylation-mediated transcriptional upregulation of glutathione S-transferases (GSTs) enhances the conjugation and clearance of cisplatin and alkylating agents^[59,60]^.

### Target alteration

Tumor cells may acquire resistance through mutation or conformational change of drug targets. While direct lactylation of mutated oncogenic proteins remains underexplored, lactylation has been shown to alter transcriptional regulation of key targets such as thymidylate synthase (TYMS), EGFR, and topoisomerase IIα. For instance, in 5-FU-resistant gastric cancer cells, lactylation-enhanced expression of TYMS sustains DNA synthesis despite drug presence. In breast cancer, lactylation of the estrogen receptor has been linked to resistance against tamoxifen^[61]^.

Taken together, these findings demonstrate that lactylation not only governs epigenetic control of classical resistance factors (transporters, detox enzymes, target genes), but also establishes an early biochemical basis for therapy evasion. Targeting lactylation in these contexts may help reverse primary resistance mechanisms before adaptive pathways (e.g., DNA repair or autophagy) are activated.

## THE IMPACT OF LACTYLATION ACCUMULATION ON TUMOR CELL DRUG RESISTANCE

### Lactylation-mediated transcriptional reprogramming of drug resistance genes

Histone lactylation functions as a crucial epigenetic mechanism in tumor cells by modulating gene expression programs associated with drug resistance. Acting in a manner analogous to acetylation, lactylation at specific lysine residues - particularly on histone H3 - alters chromatin conformation to enhance transcriptional activity. This section categorizes lactylation-induced transcriptional reprogramming into three major phenotypic outputs: enhanced metastasis and invasion, immune evasion, and upregulation of drug resistance-associated genes.

### Upregulation of metastasis- and EMT-related genes

Lactylation, as an epigenetic modification, primarily functions by directly regulating gene expression^[62]^. Histone lactylation alters chromatin conformation and gene transcriptional activity. Similar to histone acetylation, lactylation typically occurs on the ε-amino group of lysine residues, neutralizing their positive charge. This reduces the affinity between histones and DNA, leading to a more open chromatin structure^[63]^. Initial research by He *et al.* demonstrated that specific sites of histone H3 lactylation in macrophages are upregulated under hypoxic or high-lactate conditions, resulting in the activation of corresponding gene transcription^[4]^. This principle similarly applies to tumor cells: elevated lactylation levels in tumor cells may drive increased expression of oncogenes or drug-resistance genes.

Lactylation promotes expression of pro-metastatic genes across several tumor types. H3K18la activates VCAM1 transcription in gastric cancer, enhancing invasion and chemotherapy resistance. In ESCC, H3K9la enrichment at the LAMC2 promoter under hypoxia promotes EMT, correlating with radiotherapy resistance^[51,64,65]^.

In HCC, H3K9la and H3K56la upregulate ESM1, a factor associated with tumor vascularization and drug tolerance^[46]^. Similarly, H3 lactylation enhances Sox9 and CENPA transcription in liver tumors, driving proliferation and resistance phenotypes^[66,67]^. In addition, non-histone lactylation is also involved in HCC progression; for example, the latest research has shown that non-histone ABCF1-K430 lactylation promotes the malignant progression of HCC through transcriptional activation of the HIF1 signaling pathway, but its relationship with drug resistance phenotype still needs to be further confirmed^[68]^.

### Immune checkpoint and immunosuppressive gene activation

Lactylation also facilitates immune evasion by enhancing transcription of immunosuppressive genes. In ovarian cancer, macrophage H3K18la upregulates CCL18, fostering an M2-polarized microenvironment resistant to immunotherapy^[58]^. In addition, lactic acid in NSCLC cells stimulates the lactation modification of non-histone APOC2-K70, promotes the breakdown of extracellular fat to produce free fatty acids (FFA), and induces resistance to tumor immunotherapy^[69]^.

In GBM, lactylation activates CD39, CD73, and CCR8, suppressing antitumor immunity^[70,71]^.

Notably, in acute myeloid leukemia (AML), STAT5 recruits acetyltransferases to induce lactylation of PD-L1 promoter regions^[72]^, upregulating PD-L1 expression and mediating immune escape under chemotherapy pressure^[73,74]^.

### Drug resistance gene activation and oncogene amplification

Multiple drug resistance-related genes are regulated by histone lactylation. In colorectal cancer, H3K18la promotes transcription of CXCL1, CXCL5, and RUBCNL, enhancing resistance to bevacizumab and 5-FU^[4]^. Lactylation also activates expression of the long non-coding RNA LINC00152^[72,75]^, METTL3, and Kcnk6, promoting multidrug resistance phenotypes^[76-78]^.

In ccRCC, lactylation of CBX3 at K10 enhances PDGFRβ transcription, contributing to VEGF-targeted therapy resistance^[65]^. In prostate cancer, lactylation of HIF-1α upregulates KIAA1199, driving angiogenesis and resistance to anti-VEGF agents^[79-81]^.

### Functional complexity and reader uncertainty

While most studies link lactylation to gene activation, emerging evidence suggests that specific lactylation sites may also repress gene transcription, depending on recruited “reader” proteins. For example, AARS1 binds directly to lactate and catalyzes site-specific lactation in the DNA-binding domain of p53, a modification that prevents p53 from binding to DNA and thus inhibits the transcriptional activity of the *p53* gene^[20]^. Bromodomain-containing proteins, known for recognizing acetylation, may similarly interact with lactylated lysines; however, this mechanism remains under investigation. Nevertheless, histone lactylation represents a critical regulatory layer in the epigenetic control of drug-resistant phenotypes.

Collectively, lactylation broadly remodels the gene expression landscape of tumor cells through dual mechanisms. First, it regulates transcription via histone modifications that alter chromatin state, influence promoter activity, and affect the function and stability of transcription factors. Second, it recruits various reader proteins that either activate or inhibit gene expression. Together, these processes promote drug resistance and enhance the survival of tumor cells.

### Enhanced DNA damage repair via lactylation

It is well known that chemotherapy and radiotherapy induce DNA damage (such as strand breaks, base mismatches, cross-links, *etc.*) as one of the important therapeutic mechanisms that trigger cancer cell death. Cellular injury repair mechanisms (such as direct repair, mismatch repair, HR and non-homologous end joining, *etc.*) promote cell survival by maintaining genome stability. In tumor cells, overactivation of the repair pathway, mutation or overexpression of repair proteins, activation of the bypass pathway, and the influence of the TME all help cancer cells resist the killing effects of chemotherapy and radiotherapy. Lactylation has been demonstrated to enhance DNA repair capacity in cancer cells^[82-84]^.

Tumor cells often exploit DNA repair mechanisms to counteract chemotherapy-induced genotoxic stress. Lactylation has emerged as a crucial regulator of HR, conferring resistance to DNA-damaging agents such as platinum compounds and PARP inhibitors.

A 2024 study published in *Nature* provided the first evidence that lactate-mediated lactylation modifications directly influence DNA damage repair pathways^[14]^. Recent studies have demonstrated that lactylation directly modifies key DNA repair proteins. Under high-lactate conditions, lysine 388 on NBS1 - a core component of the MRN complex (MRE11-RAD50-NBS1) - undergoes lactylation, facilitating complex formation and HR repair efficiency. Inhibition of NBS1-K388 lactylation impairs DNA repair and sensitizes tumor cells to cisplatin. Similarly, lactylation at lysine 673 of MRE11 enhances DNA binding and end resection activity, further promoting repair fidelity^[85]^.

This lactylation-mediated enhancement is regulated by epigenetic modifiers: the acetyltransferase Tip60 (KAT5) acts as a “writer” of NBS1 lactylation, while HDAC3 functions as an “eraser”. Clinically, elevated levels of NBS1-K388la are associated with poor responses to neoadjuvant chemotherapy and unfavorable prognosis in breast and ovarian cancers^[14,82]^.

Together, these findings define a critical resistance axis - lactate–lactylation–NBS1/MRE11–HR repair - which enables tumor cells to rapidly restore genomic integrity following therapeutic insult. Targeting this axis through lactylation inhibition may restore DNA damage sensitivity and improve chemotherapy efficacy.

### Activation of autophagy pathways via lactylation

Autophagy is a conserved cellular process that enables tumor cells to degrade and recycle intracellular components, providing metabolic support and resistance to therapeutic stress. Emerging evidence has revealed that lactylation acts as a key modulator of autophagy, thereby promoting drug resistance in various cancers.

Mechanistically, lactylation enhances autophagic flux through both transcriptional and post-translational regulation. One pivotal event involves lactylation at lysine 91 (K91) of transcription factor EB (TFEB), which disrupts its interaction with the E3 ubiquitin ligase WWP2. This prevents TFEB ubiquitination and proteasomal degradation, leading to its nuclear translocation and sustained activation of lysosomal biogenesis and autophagy-related gene expression^[83,84]^.

At the protein complex level, lactylation of PIK3C3/VPS34 enhances its lipid kinase activity by stabilizing its interaction with autophagy scaffold proteins such as BECN1, ATG14, and UVRAG. This facilitates endolysosomal degradation and promotes tumor cell survival under chemotherapeutic pressure. Additionally, lactylation stabilizes RUBCNL/PACER, a regulator that represses RIPK1-dependent apoptosis and necroptosis, further supporting tumor persistence^[86-88]^.

Beyond transcriptional effects, lactylation influences autophagy signaling cascades. Lactate-driven lactylation may activate the AMPK-mTOR axis and induce autophagy initiators such as ULK1^[89,90]^. Furthermore, modification of autophagy-related proteins (e.g., ATG5) enhances their stability and function, accelerating autophagosome formation and degradation of damaged organelles^[91,92]^.

Therapeutically, these findings highlight the potential of targeting lactylation-autophagy interactions. Combining lactylation inhibitors with late-stage autophagy blockers (e.g., chloroquine) may restore drug sensitivity in resistant tumors. Future studies are needed to delineate the full scope of lactylation’s role in autophagy and its translational implications in oncology.

### Activation of pro-survival signaling pathways via lactylation

Lactylation modulates multiple oncogenic signaling cascades, thereby reinforcing drug resistance by promoting tumor cell proliferation, survival, and immune evasion. Several key pathways - including Wnt/β-catenin, JAK/STAT3, PI3K/AKT, NF-κB, Hippo, and TGF-β - have been implicated in lactylation-driven therapeutic resistance.

In colorectal cancer, hypoxia-induced lactylation of β-catenin enhances its transcriptional activity and activates the Wnt pathway, promoting cancer stemness and resistance to oxaliplatin^[49]^. Similarly, lactylation of histones in macrophages downregulates RARγ, triggering TRAF6-mediated NF-κB signaling and IL-6 production, which in turn activates STAT3 - a known driver of chemoresistance^[93,94]^.

In head and neck squamous cell carcinoma, histone H3K9 lactylation upregulates IL-11 expression, reinforcing JAK2/STAT3 signaling and immunosuppressive phenotypes, leading to poor therapeutic outcomes^[95]^. In GBM stem cells, lactylation-induced expression of MAP4K4 activates the JNK pathway, sustaining self-renewal capacity under chemotherapeutic stress. In endometrial carcinoma, upregulation of USP39 via H3K18la activates PI3K/AKT/HIF-1α signaling, promoting survival and angiogenesis^[96,97]^.

Lactylation has also been shown to suppress tumor-suppressive pathways. For instance, in bladder cancer, histone lactylation inhibits the expression of circXRN2, a regulator of the Hippo pathway, thereby facilitating tumor progression^[98]^. Moreover, lactylation of MOESIN enhances regulatory T cell (Treg) expansion through TGF-β signaling, supporting immune escape and resistance to immunotherapy^[99,100]^.

**Figure 2:**
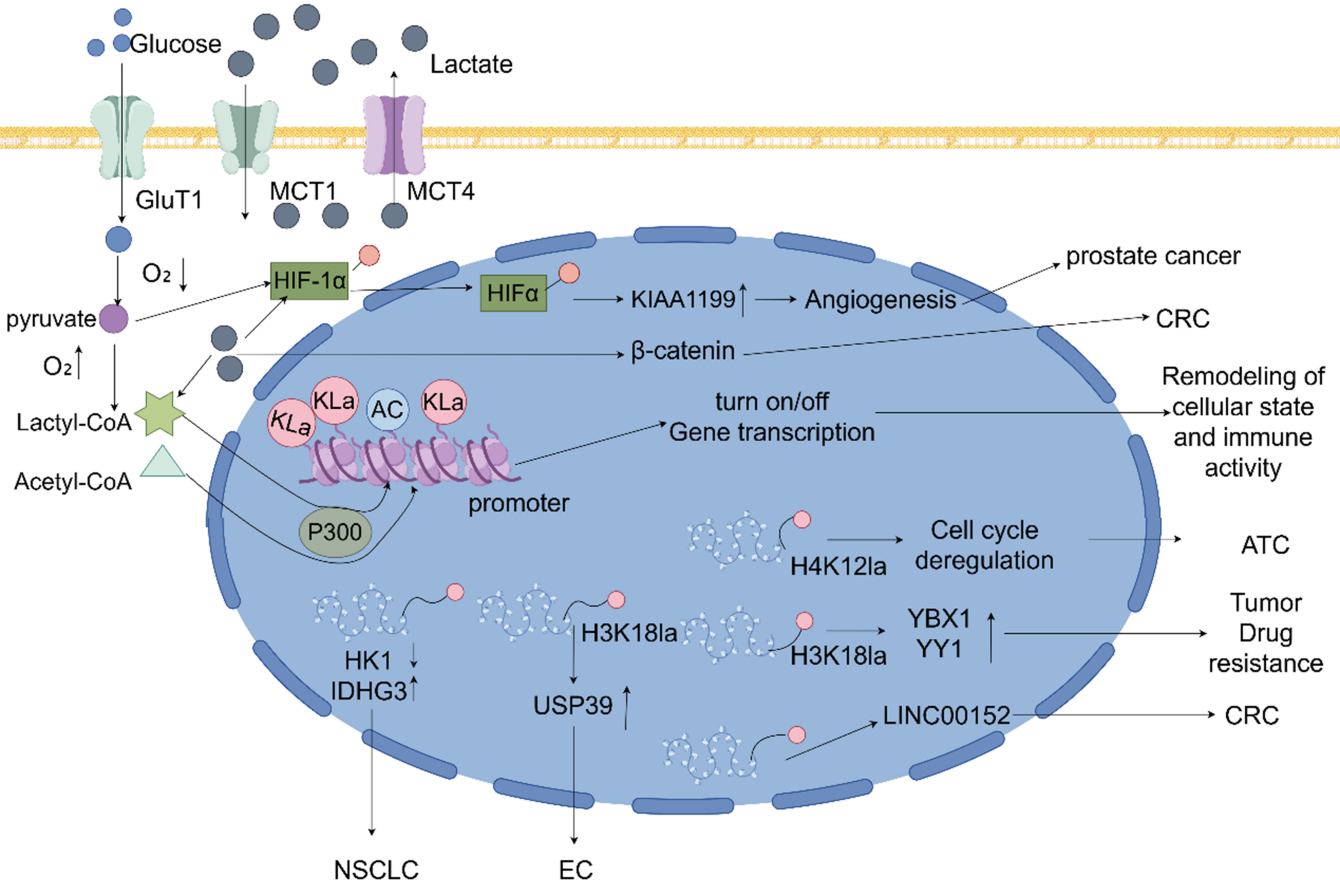
Lactate as a signaling molecule influencing gene transcription and immune evasion through histone and non-histone lysine lactylation, promoting cancer progression. Glycolysis breaks down glucose into pyruvate. Under hypoxic conditions, lactylation of HIFα is activated, which subsequently promotes the expression of KIAA1199, angiogenesis, and the progression of prostate cancer. Under aerobic conditions, lactyl-CoA and acetyl-CoA, driven by P300, catalyze lactylation and acetylation of promoters, thereby reshaping cellular states and immune activity by upregulating or downregulating gene transcription. Elevated levels of H4K12la disrupt the cell cycle and accelerate the progression of ATC. H3K18 lactylation not only enhances the expression of transcription factors YBX1 and YY1 to promote tumor drug resistance but also upregulates USP39 to facilitate the progression of EC. Histone lactylation at the LINC00152 promoter, combined with β-catenin lactylation, jointly drives CRC progression. Moreover, lactylation promotes NSCLC progression by downregulating HK1 expression and upregulating IDHG3 expression. GluT1: Glucose transporter 1; MCT1/4: monocarboxylate transporter 1/4; Kla: histone lysine lactylation; AC: acetylation; HIF: hypoxia-inducible factor; HK1: hexokinase 1; IDHG3: isocitrate dehydrogenase 3; USP39: ubiquitin-specific protease 39; ATC: anaplastic thyroid cancer; NSCLC: non-small cell lung cancer; CRC: colorectal cancer; EC: endometrial cancer.

These findings underscore the multifaceted role of lactylation in reprogramming oncogenic signaling networks. Therapeutic strategies that block lactylation-sensitive nodes - such as STAT3 or PI3K/AKT - may restore drug efficacy and suppress resistance-related signaling [Figure 2].

### Lactylation-mediated immunosuppression and tumor drug resistance

**Figure 3:**
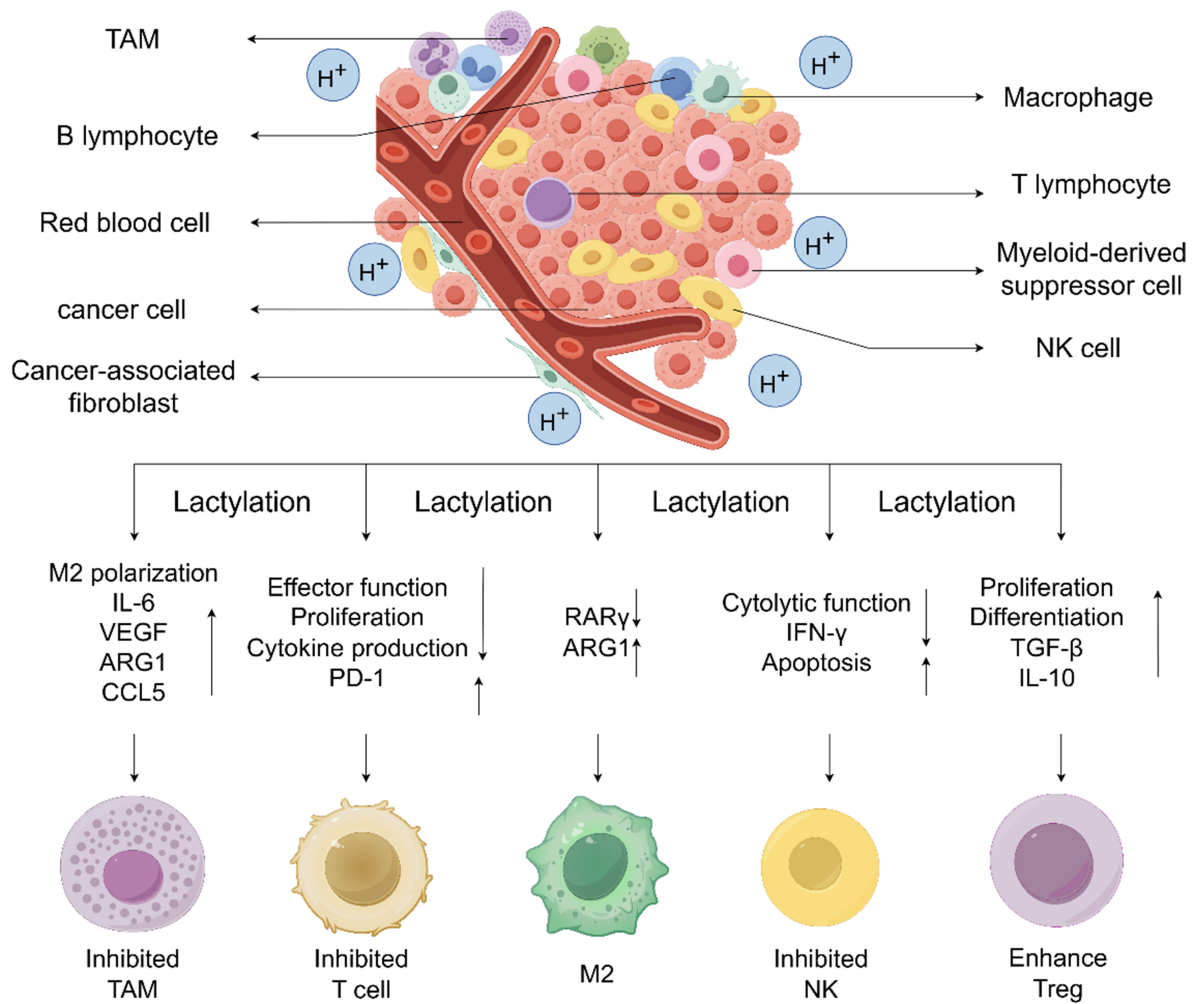
In tumor cells, lactylation regulates the function of immune cells within the TME, contributing to immune evasion and tumor progression. Lactylation has been shown to suppress the cytotoxic and antitumor effects of IFN-γ by promoting apoptosis and impairing NK cell function. This diminishes the immune system’s ability to target and eliminate tumor cells. Furthermore, lactylation enhances the expression of the immune checkpoint molecule PD-1, resulting in the suppression of cytokine production, proliferation, and effector functions of T cells. This leads to the attenuation of T cell-mediated immune responses, thereby facilitating tumor growth. Lactylation also drives the polarization of TAMs from an antitumorigenic M1 phenotype to a pro-tumorigenic M2 phenotype. This is achieved through upregulation of ARG1 and downregulation of RARγ. Increased production of IL-6, VEGF, ARG1, and CCL5 further suppresses the phagocytic activity of TAMs, aiding tumor progression. In addition, lactylation enhances the proliferation and differentiation of Tregs by boosting the expression of TGF-β and IL-10. This strengthens the immunosuppressive function of Tregs, enabling tumor cells to evade immune surveillance and promoting tumorigenesis. IFN-γ: Interferon-gamma; RARγ: retinoic acid receptor gamma; PD-1: programmed cell death protein-1; IL-6: interleukin-6; VEGF: vascular endothelial growth factor; ARG1: arginase-1; CCL5: chemokine ligand 5; TGF-β: transforming growth factor-beta; IL-10: interleukin-10.

Lactylation promotes tumor immune evasion by modulating immune cell function, regulating key immunomodulatory proteins, and altering RNA modifications within the TME [Figure 3].

In macrophages, lactylation drives polarization toward the pro-tumoral M2 phenotype. In prostate cancer models, it suppresses TAM activity and facilitates tumor progression^[38,101,102]^. In colorectal cancer, lactylation of retinoic acid-inducible gene I (RIG-I) and suppression of RARγ synergistically enhance M2 polarization and immune suppression^[93,94]^.

Lactylation of H3K9 and H4K5 suppresses CD8^+^ T cell function^[74,95]^. Neutrophil cytotoxicity is also impaired by lactylation, leading to enhanced metastatic potential. In natural killer (NK) cells, lactylation directly inhibits cytotoxicity and indirectly reduces NK populations by expanding myeloid-derived suppressor cells (MDSCs)^[103-105]^. Moreover, MOESIN lactylation promotes Treg development and function, reinforcing immunosuppression in the TME.

Beyond immune cells, lactylation influences tumor immune escape via RNA methylation. In tumor-infiltrating myeloid cells (TIMs), lactylation enhances colorectal cancer progression through METTL3-mediated m^6^A RNA modification^[78,106]^. In gastric cancer, copper-induced lactylation of METTL16 enhances tumorigenesis via similar m^6^A-dependent pathways^[107]^. In addition, circATXN7 expression, activated by lactylation, promotes immune escape through NF-κB signaling^[108]^.

Given its central role in immune evasion, targeting lactate metabolism and lactylation holds therapeutic promise. Inhibition of lactate dehydrogenase (LDH) has been shown to suppress both tumor growth and immune escape^[109,110]^.

### Lactylation-mediated angiogenesis and tumor drug resistance

Tumor angiogenesis plays a central role in cancer progression by supplying oxygen and nutrients and facilitating metastasis. Lactylation has been shown to promote angiogenesis through multiple mechanisms, thereby contributing to therapeutic resistance^[111,112]^.

Hypoxia-induced expression of HIF-1α, VEGF, and IL-8 initiates angiogenesis in tumors. Lactate enters endothelial cells via MCT-1, activating the NF-κB/IL-8 axis and upregulating VEGF expression^[113,114]^. Compared to lactate itself, histone lactylation appears to exert more direct pro-angiogenic effects.

For example, in HCC, lactylation activates the JAK2/STAT3 pathway and upregulates GP73, enhancing angiogenesis^[115]^.

In prostate cancer, histone lactylation elevates KIAA1199 expression, further promoting neovascularization and resistance to anti-angiogenic therapies^[81,116]^. In colorectal cancer, histone lactation enhances the expression of the oncogene RUBCNL and promotes resistance to bevacizumab, a specific targeted therapy for anti-angiogenic genesis, in CRC^[89]^.

Initial therapeutic strategies have emerged. The natural compound evodiamine inhibits HIF1A lactylation, blocks angiogenesis, and restores ferroptosis sensitivity in tumors^[117]^.

### Lactylation promotes chemoresistance in tumors

Chemoresistance remains a major obstacle to successful cancer treatment. Accumulating evidence indicates that lactylation contributes to chemoresistance through multiple mechanisms^[118,119]^.

In brain metastases of lung cancer, AKR1B10 upregulates H3K18 lactylation, suppresses LDHA expression, and induces resistance to PEM^[17]^.

Conversely, elimination of CNPY3 lactylation improves therapeutic outcomes^[120]^.

In GBM, lactylation activates LUC7L2 transcription, promoting resistance to TMZ^[121]^. Zhu *et al.* developed an optimized Riskscore model of lactation from TCGA and GEO databases and identified a significant association between lactation and chemoresistance in patients at high risk of lymphoma^[122]^. In bladder cancer, single-cell RNA sequencing reveals that lactylation-driven activation of transcription factors YBX1 and YY1 underlies cisplatin resistance^[123,124]^.

Lactylation also contributes to chemoresistance by suppressing ferroptosis. In HCC and colorectal cancer stem cells, H3K18la and H4K12la inhibit lipid peroxidation and enhance treatment resistance^[125,126]^. HDAC inhibitors and shikonin restore ferroptosis sensitivity and enhance chemotherapy efficacy^[127,128]^.

In summary, lactylation drives chemoresistance through transcriptional regulation, pathway activation, and ferroptosis suppression, highlighting its value as a therapeutic target.

Multiple genetic loci associated with histone lactylation promote the development of various tumor cells to varying degrees [Table 2].

**Table 2:** Roles of lactate modifications in cancer cells

**Cell type**	**Lactylated protein(s)/site(s)**	**Function**	**Ref.**
PCa cell	H3K18	Promotes neuroendocrine differentiation	[129]
HCC cell	CCNE2	Promotes tumor proliferation	[130]
	H3K56	Promotes tumorigenesis	[131]
	H3K18	Promotes tumor proliferation	[132]
NSCLC cell	H3	Inhibits glycolysis	[133]
GBM cell	H3K9la	Activates LUC7L2 transcription	[121]
OM cell	H3K18	Upregulates the oncogene YTHDF2 and facilitates degradation of PER1 and TP53 mRNAs	[134]
ccRCC cell	H3K18	Activates PDGFRβ transcription and promotes ccRCC progression	[40]
BMDM cell	H3K18,23	Promotes tumor proliferation	[38]
LCSCs	H3K56	Promotes HCC proliferation	[131]
	H3K18	Facilitates SOX9 transcription	[67]
TIMs	H3K18	Promotes CRC and upregulates METTL3	[77]
ESCC cell	H3K9	Enhances LAMC2 transcription	[51]
CRC cell	H3K18	Promotes expression of autophagy enhancer protein RUBCNL	[89]
GC cell	H3K18	Mediates VCAM1 expression and promotes GC progression	[64]

PCa: Prostate cancer; HCC: hepatocellular carcinoma; NSCLC: non-small cell lung cancer; GBM: glioblastoma; OM: ocular melanoma; ccRCC: clear cell renal cell carcinoma; BMDM: bone marrow-derived macrophage; LCSCs: liver cancer stem cells; TIMs: tumor-infiltrating myeloid cells; ESCC: esophageal squamous cancer; CRC: colorectal cancer; GC: gastric cancer.

## LACTYLATION MODIFICATION: AN EMERGING THERAPEUTIC TARGET FOR TUMOR DRUG RESISTANCE

Emerging studies have identified lactylation as a key epigenetic and metabolic mechanism promoting drug resistance, immune escape, and tumor progression. Therapeutically targeting the lactylation axis - either by inhibiting lactate production, blocking lactyltransferases, or disrupting lactate transport - offers a multifaceted strategy to suppress resistance pathways. This section summarizes targeted approaches and their mechanistic links to lactylation in cancer.

### Lactate production: inhibition of LDHA and LDHB

LDH, especially LDHA, catalyzes the conversion of pyruvate to lactate and provides the substrate for histone lactylation. High LDH expression is associated with elevated tumor lactate levels and increased lactylation in drug-resistant phenotypes. Clinical evidence correlates elevated LDH levels with poor responses to PD-1/PD-L1 immunotherapy and unfavorable prognosis in melanoma and lung cancer patients^[81,117,118]^. Selective LDHA inhibitors (e.g., FX11, GNE-140, GSK2837808A) reduce intracellular lactate and suppress lactylation modifications. Consequently, they restore chemosensitivity^[135-138]^.

Although most research focuses on LDHA, recent data suggest that LDHB silencing also reduces lactate flux and tumor cell viability, indicating its complementary role in targeting lactate-driven resistance^[139]^.

### Targeting the “Writer” enzyme: P300 inhibitors

The histone acetyltransferase P300 has recently been identified as a lactyltransferase that directly installs lactate groups onto histone lysine residues, particularly H3K18 and H3K9. Overexpression of P300 correlates with poor outcomes in HCC, prostate cancer, and lymphoma^[140-142]^. Small-molecule P300 inhibitors (e.g., CCS1477, A-485) block lactylation at key resistance-associated gene loci, including PD-L1 and VCAM1. This inhibition reverses chemoresistance and reduces EMT signatures^[143-145]^. Notably, combining P300 inhibitors with immunotherapy (e.g., anti-PD-1 agents) has demonstrated enhanced efficacy in preclinical models, highlighting potential for combinatorial strategies.

### Targeting glycolytic metabolism to reduce lactylation substrates

Given that glycolysis fuels lactate accumulation and thereby lactylation, metabolic inhibitors offer indirect - but effective - strategies to modulate lactylation. 2-Deoxy-D-glucose (2-DG), a hexokinase inhibitor, reduces glycolytic flux and lactate output, leading to decreased H3 lactylation and suppressed tumor growth^[146]^.

Natural compounds such as Fargesin, demethylzeylasteral (DML), and tanshinone I inhibit lactylation through glycolysis blockade, particularly in liver, lung, and ovarian cancer models. Royal jelly acid (RJA) specifically reduces H3K9la and H3K14la in HCC, inhibiting downstream gene expression involved in resistance and angiogenesis^[147-149]^.

### Inhibition of lactylation-activated signaling pathways

Several resistance-related pathways are activated downstream of transcription factors modified by lactylation. Targeting these pathways may synergize with lactylation inhibition.

### PI3K/AKT/mTOR pathway

Targeting specific signaling pathways to interfere with lactylation has also demonstrated significant antitumor potential. Even before the identification of lactylation modifications, researchers found that disrupting the PI3K/AKT/mTOR signaling pathway effectively inhibited lactate metabolism utilization^[149-151]^. Everolimus, for example, suppresses the progression of various tumors via this mechanism^[152-155]^.

These insights support the utility of pathway-targeted agents in combination with lactylation inhibitors.

### PDGFRβ and JAK/STAT3 pathways

Recent studies have further confirmed that inhibition of lactylation can block the PDGFRβ pathway, enhancing therapeutic efficacy in ccRCC^[40]^. Additionally, suppression of the JAK/STAT3 pathway can improve prognosis in HCC. In mouse models of prostate cancer, blocking lactylation-mediated signaling pathways significantly suppressed tumor growth, highlighting substantial therapeutic promise^[102,115,156]^.

### Blocking lactate transport: targeting MCT1 and MCT4

These insights support the utility of pathway-targeted agents in combination with lactylation inhibitors^[157,158]^. AZD3965, an MCT1 inhibitor, has shown promising results in blocking lactate uptake and reducing lactylation-driven gene expression. Fluvastatin, recently identified as an MCT4 inhibitor, disrupts lactate export and induces metabolic stress in lung adenocarcinoma models^[159-162]^. Both agents impair lactylation homeostasis and re-sensitize tumors to chemotherapy^[143]^.

### Future perspectives and clinical translation

Beyond monotherapy, future strategies may combine lactylation-targeted drugs with immunotherapies, ferroptosis inducers, or autophagy inhibitors. Additionally, lactylation-specific biomarkers (e.g., H3K18la, NBS1-K388la) may aid in patient stratification and real-time treatment monitoring. Nanotechnology-based drug delivery platforms, such as LDHA-inhibitor-loaded nanoparticles, are under development to enhance tumor targeting and minimize systemic toxicity.

Table 3 summarizes representative studies demonstrating the application of nanoformulations to modulate tumor lactate metabolism and enhance immunotherapeutic efficacy.

**Table 3:** Application of nanoformulations in lactated pharmacodynamic studies of tumors

**Nanoformulation**	**Model**	**Mechanism of intervention**	**Lactate level changes**	**Pharmacodynamic effects**	**Ref.**
Lactate oxidase nanocapsules	Mouse melanoma model; humanized mouse model of triple-negative breast cancer	Nanocapsules deliver lactate oxidase, reducing lactate levels and promoting hydrogen peroxide production	Decreased lactate levels	Enhances T cell immunity, overcomes tumor-induced immunosuppression, and improves the efficacy of immune checkpoint blockade therapy	[163]
Nanoparticle loaded with lactate oxidase and Ce@ZIF-8	*In vitro*: HEPA1-6 cells; *in vivo*: HCC mouse model	Oxidizes lactic acid within tumors, producing ·OH *in situ* to induce tumor cell apoptosis, mitochondrial damage, and activation of M1 macrophages and CD8^+^ T cells	Lactate depletion in tumors	Enhances antitumor immunity through metabolic reprogramming and immune remodeling, thereby improving immunotherapy efficacy	[164]
Alkaline sodium bicarbonate nanoparticles (NaHCO_3_ NPs)	4T1 mouse breast cancer cells; breast cancer mouse model	Neutralizes the acidic TME, regulates lactate metabolism, releases Na^+^ ions to increase intracellular osmotic pressure, activates apoptosis, and improves immune response	Reduced lactate levels	Inhibits primary and metastatic tumor growth, activates immune responses, and enhances antitumor immunity	[165]
PBNM-based nanocatalyst integrated with MCT4 and β-lapachone	HUH-7 (HCC cells), 4T1 cells; Huh-7 tumor mouse model	Disrupts lactate transport to regulate local pH, selectively increases ROS in tumor cells, blocks lactate efflux, promotes hydroxyl radical formation, and activates redox cycling and H_2_O_2_ regeneration	Decreased lactate levels	Inhibits tumor growth, metastasis, and cancer stemness without causing significant toxicity to normal cells	[166]
SYR/LOD@HFN nano platform	B16-F10 melanoma cell line	SYR inhibits lactate efflux; LOD catalyzes H_2_O_2_ production. Resulting ROS damage mitochondria, suppress oxidative phosphorylation, induce proinflammatory cytokines, restore effector T and NK cells, increase M1 macrophage polarization, and suppress Tregs via MCT1/MCT4 blockade	Reduced tumor lactate levels	Reverses pH gradient and remodels the antitumor immune microenvironment. Enables synergistic chemodynamic, immune, and starvation therapies, significantly enhancing antitumor efficacy	[167]
HMONs@HCPT-BSA-PEI-CDM-PEG@siMCT-4 nanoplatform	B16F10 and 4T1 tumor mouse models	Silencing MCT-4 expression inhibits lactate efflux, promotes M1 macrophage polarization, restores CD8^+^ T cell activity, enhances immune infiltration, and induces tumor cell apoptosis	Decreased tumor lactate levels	Alleviates immunosuppressive TME, activates immune response, suppresses tumor growth and lung metastasis, and enhances chemoimmunotherapy	[168]

Table 3 highlights the application of various nanoformulations in lactate-targeted pharmacodynamic tumor studies. Each formulation is shown to modulate lactate levels in tumor cells through different mechanisms in various animal models, ultimately enhancing antitumor chemoimmunotherapy outcomes. HCC: Hepatocellular carcinoma; TME: tumor microenvironment; NK: natural killer; MCT1/4: monocarboxylate transporter 1/4.

**Figure 4:**
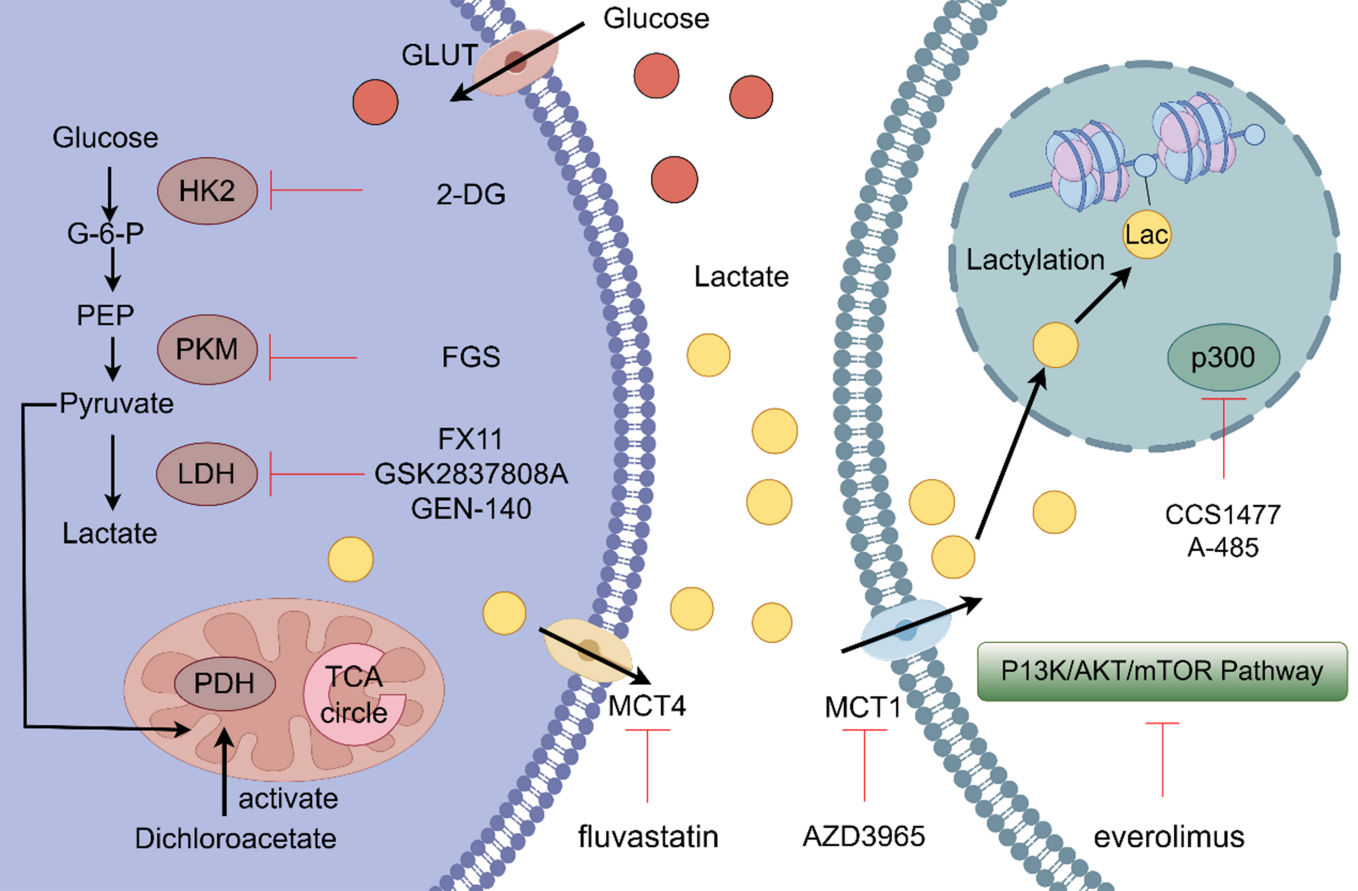
Certain drugs and genetic materials influence the process of lactate metabolism by targeting various key points in the pathway. 2-DG competitively inhibits HK2 by binding to its relevant site, thereby preventing the interaction between HK2 and glucose, ultimately reducing lactate production. FGS acts on PKM, suppressing lactate generation in tumor cells. GSK2837808A, FX11, and GEN-140 inhibit the conversion of pyruvate to lactate by modulating the expression and activity of LDH. Dichloroacetate activates PDH, promoting the TCA cycle and thereby inhibiting the conversion of pyruvate to lactate. Fluvastatin targets the transporter MCT4, suppressing lactate export. AZD3965 acts on the transporter MCT1, thereby inhibiting the cellular uptake of lactate, disrupting lactate metabolism. Everolimus, an mTOR inhibitor, suppresses the PI3K/AKT/mTOR signaling pathway in tumor cells and consequently inhibits cancer progression. 2-DG: 2-Deoxy-D-glucose; HK2: hexokinase 2; PKM: pyruvate kinase M; LDH: lactate dehydrogenase; PDH: pyruvate dehydrogenase; FGS: fargesin; MCT1/4: monocarboxylate transporter 1/4; TCA: tricarboxylic acid.

Overall, targeting the lactylation axis represents a promising and multifaceted approach to overcoming tumor resistance and improving cancer therapy outcomes. Several inhibitors targeting this pathway have already advanced to clinical trials [Figure 4 and Table 4].

**Table 4:** Onco-therapeutic drugs targeting lactylation

**Target**	**Drug name**	**Mechanism/application**	**Ref.**
LDHA/B	GSK2837808A	Inhibits LDHA activity to exert antitumor effects	[137,138,169]
LDHA/B	Galloflavin	Binds to catalytic site and reduces lactate generation	[170]
LDHA/B	N-hydroxyindole	Disrupts lactate metabolism and indirectly inhibits p300 lactyltransferase	[171]
LDHA	FX11	Selectively inhibits LDHA, reduces lactate production, and suppresses tumor growth	[135]
LDHA	GNE-140	Small-molecule LDHA inhibitor that lowers lactate production and inhibits tumor progression	[136]
PDHK	DCA	Inhibits LDHA activity to suppress lactate production and reduce drug resistance	[172,173]
P300	CCS1477	Inhibits P300 expression in tumor cells to prevent tumor progression	[143]
P300	A-485	Suppresses P300 to inhibit tumor growth	[144]
PKM2	FGS	Inhibits glycolysis and downregulates histone H3 lactylation to inhibit tumor growth	[148]
HK (Hexokinase)	2-DG	Targets glycolysis enzymes to reduce lactate accumulation and protein lactylation	[174,175]
H3 histone	DML	Inhibits the development and proliferation of M3 LCSCs	[131]
H3K9/H3K14	RJA	Disrupts glycolysis and inhibits HCC progression	[147]
PI3K/AKT/mTOR pathway	Everolimus	Blocks this signaling pathway to inhibit multiple cancer types	[152,154,155,176]
MCT1	AZD3965	Inhibits MCT1, activates AMPK, and promotes ferroptosis in HCC	[162]
MCT4	Fluvastatin	Disrupts lactate homeostasis and inhibits lung adenocarcinoma growth	[177]

LDHA/B: Lactate dehydrogenase A/B; PKM2: pyruvate kinase M2; 2-DG: 2-Deoxy-D-glucose; DML: demethylzeylasteral; LCSCs: liver cancer stem cells; RJA: royal jelly acid; HCC: hepatocellular carcinoma; MCT1/4: monocarboxylate transporter 1/4.

## CONCLUSION

Lactylation, acting as a pivotal epigenetic integrator linking metabolic reprogramming and epigenetic regulation, systematically translates key features of the TME - notably hypoxia, inflammation, and glycolytic metabolism - into multifaceted mechanisms driving therapeutic resistance in various solid tumors^[178]^. Lactate accumulation, fueled by these TME stressors, serves as the substrate for lactylation modifications on both histones (e.g., H3K9la, H3K18la, H4K12la) and non-histone proteins (e.g., NBS1, MRE11, TFEB, β-catenin, STAT5). This establishes a core axis - lactate–lactylation–functional proteins/histones–signaling pathways. The functional consequences of lactylation critically reprogram cancer cells by: (i) activating transcriptional programs for oncogenes, EMT/metastasis factors, immune checkpoints (e.g., PD-L1), and drug resistance genes; (ii) enhancing DNA damage repair capacity; (iii) promoting pro-survival autophagy; and (iv) facilitating immunosuppression and angiogenesis. Consequently, lactylation establishes a self-reinforcing network that sustains the activation of pro-metastatic, immune-evasive, and DNA repair processes, conferring substantial survival advantages and enabling tumors to evade diverse therapeutic pressures. We therefore underscore that lactylation is not merely a correlative biomarker but a fundamental functional driver deeply embedded within the core resistance machinery of cancer cells. However, its net impact and the specific pathways engaged are highly context-dependent, influenced by tumor type, genetic background, spatiotemporal TME dynamics, and therapeutic pressure. Addressing the challenges of therapeutic targeting - specificity, delivery, and evasion of resistance - and moving beyond correlative studies to establish causal mechanisms and delineate lactylation’s hierarchy within the complex resistance network remain critical for translating this knowledge into effective personalized clinical interventions targeting lactylation.

In the future, therapeutic strategies targeting lactylation hold promise as innovative approaches for overcoming tumor resistance^[179,180]^. On one hand, inhibitors targeting LDH^[181]^, histone acetyltransferase P300^[182]^, or monocarboxylate transporters (MCTs) could effectively reduce lactate levels and subsequent lactylation, thereby reversing resistant phenotypes^[183]^. On the other hand, combining lactylation-targeted interventions with immunotherapy, autophagy inhibitors, or molecular targeted therapies may further enhance therapeutic efficacy. Additionally, lactylation-specific biomarkers, such as H3K18la and NBS1-K388la, may be employed to assess individual patients’ risk of drug resistance and guide personalized treatment decisions. Future research efforts should focus on identifying lactylation-specific targets, unraveling their upstream and downstream regulatory networks, and accelerating clinical translation of lactylation-based therapeutics, ultimately providing novel breakthroughs for cancer treatment.

While significant progress has been made in understanding lactylation’s role in TME-driven drug resistance, critical knowledge gaps remain. Firstly, the precise enzymatic machinery governing site-specific non-histone lactylation, particularly the identification of dedicated “writers”, “erasers”, and “readers” beyond P300/CBP and AARS1, is largely undefined. Understanding the specificity and regulation of these enzymes for different substrates is crucial. Secondly, the dynamic interplay and potential hierarchy between lactylation and other PTMs (e.g., acetylation, methylation, ubiquitination) in regulating key resistance pathways requires systematic investigation using multi-omics approaches. Thirdly, the temporal dynamics of lactylation modifications during the evolution of drug resistance, especially in response to therapy, and their heterogeneity across different cell types within the TME (e.g., cancer cells, CAFs, TAMs, T cells) are poorly characterized. Advanced spatial transcriptomics and proteomics are required. These should be integrated with time-course studies in relevant models. Fourthly, while preclinical data targeting lactylation pathways (LDH, MCTs, P300) are promising, translating these findings into clinically viable strategies faces hurdles. Key challenges include developing isoform/site-specific inhibitors with minimal off-target effects, identifying robust predictive biomarkers for patient stratification (beyond bulk lactylation levels), and understanding potential compensatory resistance mechanisms. Future research should prioritize: (1) Comprehensive mapping and functional validation of lactyltransferases, delactylases, and lactylation-specific reader proteins; (2) Elucidation of lactylation crosstalk with other PTMs using integrated proteomics; (3) Application of single-cell and spatial technologies to resolve lactylation heterogeneity and dynamics *in situ* within the TME during treatment; (4) Development and rigorous preclinical testing of next-generation, selective lactylation pathway inhibitors; and (5) Design of clinical trials incorporating lactylation biomarkers to evaluate targeted therapies, potentially in combination with existing modalities like immunotherapy or chemotherapy. Addressing these gaps will be essential to fully harness the therapeutic potential of targeting lactylation to overcome multifactorial tumor drug resistance.

## DECLARATIONS

### Copyright

© The Author(s) 2025.
